# Development and Validation of a Multiplex Real-Time PCR Assay for Rapid Screening of Main Carbapenemase Genes in Clinical Isolates and Surveillance Samples

**DOI:** 10.3390/antibiotics14040363

**Published:** 2025-04-01

**Authors:** Francisco Javier Chamizo-López, José Gutiérrez-Fernández, María Dolores Rojo-Martín, Ana Belén Borrego-Alcaide, Alba González-Hevilla, Ana Lara-Oya, Begoña Palop-Borrás, José María Navarro-Marí, Mercedes Pérez-Ruiz

**Affiliations:** 1 Servicio de Microbiología, Hospital Regional Universitario de Málaga, Carlos Haya, s/n. 29010 Málaga, Spain; franciscoj.chamizo.sspa@juntadeandalucia.es (F.J.C.-L.); md.rojo.sspa@juntadeandalucia.es (M.D.R.-M.); abelen.borrego.sspa@juntadeandalucia.es (A.B.B.-A.); alba.gonzalez.sspa@juntadeandalucia.es (A.G.-H.); mariap.palop.sspa@juntadeandalucia.es (B.P.-B.); 2 Department de Microbiología, Universidad de Granada, Fuerzas Armadas, s/n. 18014 Granada, Spain; josegf@ugr.es; 3 Servicio de Microbiología, Complejo Hospitalario de Jaén, Av. del Ejército Español, 10, 23007 Jaén, Spain; ana.lara.sspa@juntadeandalucia.es; 4 Instituto Biosanitario de Granada, Avda, Servicio de Microbiología, Hospital Universitario Virgen de las Nieves, Fuerzas Armadas, s/n. 18014 Granada, Spain; josem.navarro.sspa@juntadeandalucia.es

**Keywords:** carbapenemases, real-time PCR, surveillance cultures, DNA extraction-free

## Abstract

Background/Objectives: Carbapenem-resistant *Enterobacterales*, largely due to carbapenemase production, are significant public health threats, which compromise treatment with key β-lactam antibiotics. Early detection is essential for guiding therapy and controlling spread. This study describes the design, optimisation and validation of a multiplex real-time PCR for the screening of the most frequent carbapenemases in our area. Methods: Primers and probes targeted at genes encoding carbapenemases *bla*_KPC_, *bla*_IMP_, *bla*_VIM_, *bla*_NDM_ and *bla*_OXA-48-group_ were designed and adapted for the development, and in silico and experimental validation of a single-tube real-time PCR. Results: A good linear correlation between the fluorescence values in the real-time PCR and the log_10_ of bacterial concentration of each carbapenemase-containing bacterial suspension was observed (R^2^ > 0.98). The limit of detection was 2–15, 16–256, 42–184, 4–42, 42–226 CFU/reaction of VIM-, IMP-, NDM-, KPC- and OXA-48-carbapenemase-containing bacteria, respectively. Intra-assay coefficient of variation for the mean Ct values ranged from 0.99% for OXA-48 to 3.34% for KPC. Inter-assay variability remained below 7%. Real-time PCR tested on bacterial isolates yielded 100% sensitivity and specificity. Analysis of rectal swabs using extracted DNA and a DNA extraction-free protocol showed good concordance with culture-based phenotypic methods. Additionally, the molecular method could detect all targets, except for one sample where only the DNA extraction-free protocol detected NDM. Conclusions: The assay offers a rapid, sensitive and specific method for the screening of major carbapenemase genes, providing an effective tool for surveillance and infection control in clinical settings. The DNA extraction-free protocol converts this method into a good alternative for screening in 24/7 clinical laboratories. Further multiplexing to target other resistance genes, on demand, could add potential benefits to this laboratory-developed method.

## 1. Introduction

Gram-negative bacteria have now become a common threat in both hospital and community settings. They are a frequent source of serious infections, including urinary tract infections, respiratory tract infections and bloodstream infections [1,2,3]. Given the increasing rise in antimicrobial resistance, treatment of these infections can be hampered. There has been an increase in resistance to both first-line and last-line antibiotics, such as carbapenems [4]. In the last update of the Bacterial Priority Pathogens List of the WHO, the carbapenem-resistant *Enterobacterales* are in the critical category [WHO Bacterial Priority Pathogens List 2024 Bacterial Pathogens of Public Health Importance, to Guide Research, Development, and Strategies to Prevent and Control Antimicrobial Resistance; https://www.who.int/publications/i/item/9789240093461 (accession date: 8 December 2024)]. Resistance to these antibiotics is often mediated by the acquisition of carbapenemases capable of hydrolysing beta-lactams, including carbapenems. These enzymes can be classified into three classes according to Ambler’s classification scheme [5]. Class A carbapenemases, known as *Klebsiella pneumoniae* carbapenemases (KPC), are commonly found in the *Enterobacterales* family, but occasionally, they can also be identified in *Pseudomonas aeruginosa* or *Acinetobacter baumannii* [6]. Class B or metallo-beta-lactamases (MBL) are characterised by requiring zinc in their active centre, and they can be inhibited by EDTA or dipicolinic acid [3]. This group includes New Delhi metallo-beta-lactamase (NDM), Verona integron-encoded metallo-beta-lactamases (VIM) and IMPases (IMP). Finally, the most common class D carbapenemases are the OXA-48-like enzymes, generally produced by *Enterobacterales* [7].

To treat a patient correctly and prevent the spread of carbapenem-resistant bacteria, the identification of these enzymes must be rapid and reliable. In recent years, several beta-lactam antibiotics have been marketed, but none, except cefiderocol, has activity against all types of carbapenemases [8]. Early knowledge of the type of carbapenemase can improve antibiotic treatment selection and increase adherence to treatment guidelines for multidrug-resistant bacteria. We can also use antibiotics such as ceftazidime–avibactam in combination with aztreonam in cases harbouring an MBL or even novel antibiotics such as cefiderocol [9,10,11,12,13]. Most of the phenotypic and molecular methods are available for the detection of carbapenemases from isolated colonies. Phenotypic assays used in clinical practice consist of the following: (a) growth-based assays that measure resistance in the presence of an antibiotic, such as the carbapenem inactivation method, (b) hydrolysis methods that detect the product catalysed by carbapenemase enzymes and (c) lateral flow immunoassays that detect carbapenemase enzymes using specific antibodies [14,15]. These assays are more time-consuming than molecular methods such as nucleic acid detection by PCR. Molecular assays provide information on the presence or absence of specific carbapenemase genes, which may have prognostic or therapeutic implications [16,17]. In addition, these methods are useful for epidemiological surveillance to assess the prevalence of carbapenemase genes in various settings [18].

In Spain, the most prevalent carbapenemases are the OXA-48 group, followed by VIM and KPC in *Enterobacterales* and VIM and IMP in *P. aeruginosa*. Since 2018, the detection of NDM has experimented with an increase in Southern Spain [19,20]. This study describes the design, optimisation and validation of a multiplex real-time PCR for the rapid and reliable detection of the most frequent carbapenemases, i.e., NDM, VIM, IMP, KPC and OXA-48.

## 2. Results

### 2.1. Real-Time PCR

Different combinations of primers/probe sets, amplification protocols and commercial master mixes were tested for the molecular screening of the five carbapenemase genes in a multiplex single-tube strategy. KPC and OXA-48 Taqman^®^ probes were labelled with 6-FAM, and the MBL Taqman^®^ probes were labelled with HEX in order to detect and differentiate these two groups of carbapenemase types.

Primers and Taqman^®^ probes concentration of 0.5 µM and 0.2 µM (OXA-48 and KPC), 1 µM and 0.4 µM (VIM, IMP and NDM) and 0.6 µM and 0.3 µM (Human RNase P), respectively, yielded the optimal conditions combined with the commercial kit Quantabio qScriptXLT 1-Step RT-qPCR ToughMix (Quantabio, Gaithersburg, MD, USA) and the P5 amplification protocol (Table 1).

### 2.2. Analytical Validation

#### 2.2.1. Efficiency of the Real-Time PCR

The real-time PCR was validated with five selected bacterial strains each containing one of the targeted carbapenemase genes.

**Figure 1:**
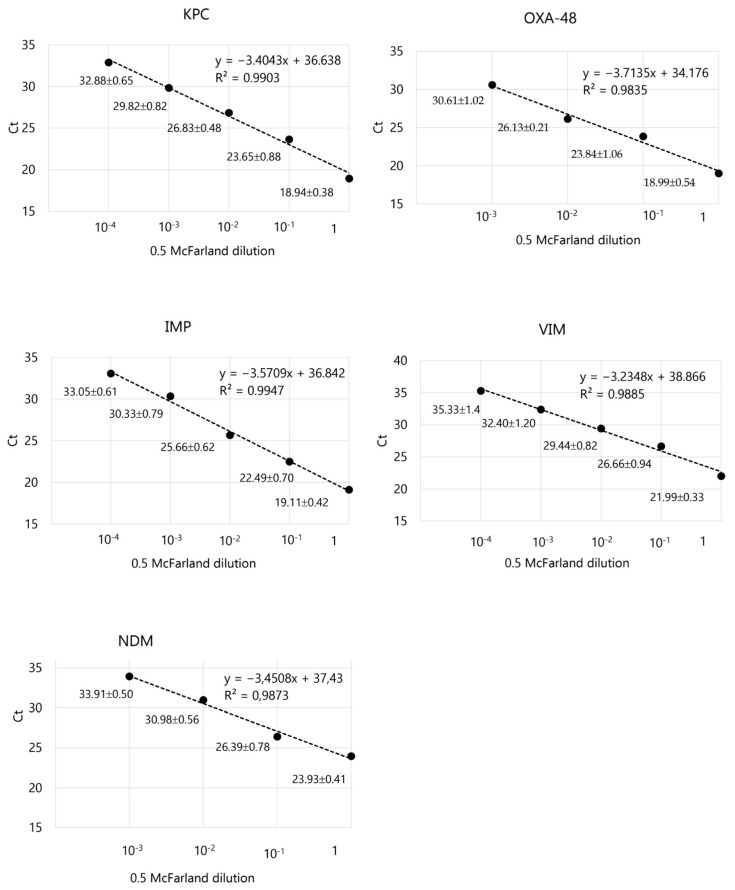
Real-time PCR correlation coefficient (R^2^) for the amplification of each carbapenemase-containing bacterial dilution of the 0.5 McFarland suspensions, within the the semi-quantitative linear range [y = Ct value; x = dilution]. Data next to the dots show mean Ct ± SD of six replicates. Bacterial strains used: *bla*_OXA-181_-positive *Klebsiella pneumoniae*, *bla*_KPC-3_-positive *K. pneumoniae*, *bla*_VIM-1_-positive *Citrobacter freundii* complex, *bla*_NDM-5_-positive *K. pneumoniae*, and *bla*_IMP-23_-positive *Pseudomonas aeruginosa*.

The real-time PCR correlation coefficient (R^2^) for the amplification of different dilutions of each carbapenemase-containing bacterial suspension was greater than 0.98 in all cases (Figure 1). These data showed a good linear correlation between the cycle threshold (Ct) values in the real-time PCR and the log_10_ of bacterial concentration of each (CFU/mL).

The limit of detection (LoD) that detected 100% of each carbapenemase gene in six replicates correlated with a real CFU count per reaction of 2–15, 16–256, 42–184, 4–42, 42–226 of VIM-, IMP-, NDM-, KPC- and OXA-48-carbapenemase-containing bacteria, respectively.

The intra-assay coefficient of variability of the mean Ct ranged from 0.99% for OXA-48 to 3.34% for KPC amplification. Inter-assay errors were under 7% in all cases and with all the dilutions tested for each carbapenemase-producing bacterium (Table 2).

#### 2.2.2. Sensitivity and Specificity

The PCR was performed on retrospective DNA from 206 bacterial isolates, 189 carbapenemases-producing isolates (72 OXA-48, 44 VIM, 35 IMP, 20 NDM, 12 KPC, 3 OXA-48 + NDM, 1 OXA-48 + VIM, 1 KPC + VIM, 1 KPC + NDM) and 17 isolates that did not produce the targeted carbapenemases. No false negative results were detected. No matches to primer or probe sequences were found other than those for the relevant genes in any of the bacterial strains tested. A 100% sensitivity and specificity were obtained when the real-time PCR was tested on bacterial isolates.

### 2.3. Molecular Screening of Carbapenemases Genes in Rectal Swabs

The real-time PCR was tested in 108 retrospective rectal swabs. A DNA extraction-free protocol was carried out in parallel with DNA extracted from the samples to evaluate whether the extraction-free method could serve as a direct assay for the molecular screening of carbapenemase-producing bacteria in surveillance programs.

All the 36 samples in which carbapenemase-producing bacteria were detected by culture were positive by PCR, except one NDM, only detected by the extraction-free protocol and one NDM detected in the purified DNA in which the direct method was invalid. In two samples, positive to NDM by phenotypic assays, MBL was detected (which would correspond to NDM) but an additional KPC/OXA-48 target was also positive with purified and extraction-free DNA (Table 3).

All samples, negative by phenotypic assays (*n* = 72), yielded a negative result in the PCR except one, in which MBL and KPC/OXA-48 were detected with purified and extraction-free DNA, with Ct values of 22 and 23.2 for the MBL target and 26.1 and 28.7 for KPC/OXA-48 target, respectively.

Ct values for the KPC/OXA-48 and MBL targets delayed a mean of 1.89 (2.62 ± 0.038) and 0.5 (2.74 ± 0.031), respectively, when extraction-free DNA was used as a template, with respect to purified DNA.

The total turnaround time of the DNA extraction-free protocol was 1.5 h compared with the 2.5 h time required when DNA extraction was carried out prior to PCR.

## 3. Discussion

To reduce healthcare-associated infections, facilities may consider screening individuals for colonisation with vancomycin-resistant enterococci (VRE) or carbapenemase-producing *Enterobacterales* (CPE). Such selective screening may be performed for high-risk hosts, including but not limited to those who are immunocompromised, undergoing dialysis, have chronic underlying conditions and frequent hospitalisations, or are contacts of infected patients [21]. In addition, recent guidelines for treating multidrug-resistant (MDR) bacterial infections emphasise the importance of considering local epidemiology and patient risk factors when selecting antimicrobial therapy [10,22,23]. These guidelines provide recommendations for managing infections caused by various MDR Gram-negative bacteria, including carbapenem-resistant *Enterobacterales*, *A. baumannii*, and *P. aeruginosa* [10,23]. The introduction of new antimicrobials, such as the new beta-lactam/beta-lactamase inhibitor combinations and cefiderocol, has widened the treatment options [10] although none of them, except cefiderocol, has activity against all types of carbapenemases.

CPE pose a significant threat in healthcare settings, necessitating effective screening methods. The rapid detection of carbapenem resistance genes can help to guide empiric antibiotic therapy, potentially improving patient outcomes in sepsis [24].

A mathematical modelling study found that while direct PCR reduced days at risk for CPE, a culture plus PCR algorithm provides optimal cost benefits and averts days at risk [25]. An in-house multiplex real-time PCR assay detected carbapenemase genes within 2.5 h, with sensitivity comparable to culture methods [26]. Economic analysis suggests that implementing PCR-based strategies upon hospital admission could lead to significant cost savings, mainly due to faster results and reduced unnecessary isolation measures [27].

With a diagnostic algorithm based on direct PCR from the rectal swab, as the one designed in the study, together with conventional culture in selective media, we would rapidly identify carriers of the most prevalent carbapenemases by PCR. With this protocol, other resistance mechanisms should be detected by conventional phenotypic methods after isolation of the microorganism, although the future challenge is to extend real-time PCR multiplexing to other targets such as VRE and extended-spectrum beta-lactamases (ESBL).

Molecular methods for detecting carbapenemase-producing organisms offer significant advantages over conventional culture techniques for surveillance and infection control. While culture-based methods are slower and less sensitive, they are more cost-effective [25,28]. Real-time PCR techniques offer rapid detection of carbapenemase genes, with shorter turnaround times compared to culture methods [24,29] and excellent sensitivity (100%) and negative predictive value (100%) for carbapenemases detection, although positive predictive value may be lower [30]. Our method could detect other carbapenemases genes in two samples, not detected by phenotypic assay.

The choice of a screening molecular method should balance cost, speed, and accuracy while considering the local epidemiological context.

Commercial PCR assays have demonstrated good performance in detecting carbapenemase genes in bacterial isolates and clinical samples. Multiple studies have reported very good sensitivity and specificity for detecting KPC, VIM, NDM and classical OXA-48 carbapenemases. Likewise, several studies have developed in-house PCR-based methods for rapid detection of carbapenemase genes in bacterial isolates [15,26,31,32,33,34,35,36,37,38,39]. Most of the in-house methods detect and identify the specific carbapenemase by real-time PCR, making necessary the use of two PCR tubes per sample or, further analysis of melting curves for specificity.

One of the most widely used commercial assays is the Xpert Carba-R PCR assay (Cepheid). However, limitations have been observed in detecting certain variants like OXA-181 and some IMP subgroups [34]. We tested the strains used for the validation of the PCR assay at the LoD calculated for our PCR assay with the Xpert Carba-R PCR. All carbapenemases were properly detected with similar efficiency to the one obtained with our PCR (equal Ct value), except for the IMP, which could not be detected with the Xpert assay. Furthermore, more concentrated suspensions of the IMP-containing strain were negative with the Xpert Carba-R PCR assay. The strain we used harboured IMP-23. Other strains containing IMP-16 and IMP-20 included in this study for the analytical validation were not detected with the Xpert Carba-R PCR assay and were efficiently detected with our assay [https://seimc.org/contenidos/congresosyeventos/seimcanteriores/seimc-congreso2018-revistaEIMC.pdf (accession date: 31 Mach 2025)].

The newer Xpert Carba-R NxG version expands detection capabilities, improving IMP detection compared to the original version and includes SPM and GES carbapenemases [40], but we have not tested our strains with this kit.

Although we have in silico demonstrated the efficiency of the designed assay to detect certain types of each of the five targeted carbapenemases, the alignment of the primers and probes chosen and the newly designed with the cabapenemases genes available, showed no mismatches. Moreover, it could detect IMP types that failed to be detected with the Xpert Carba-R.

We have chosen a single-tube protocol, which limits the number of targets that can be differentiated by fluorescence reading, considering it an optimal strategy for molecular surveillance purposes and for directing antibiotic treatment when detected from infection. It uses two fluorescence channels for KPC/OXA-48 carbapenemase and B1 MBL and a third one for internal control detection. It frees at least two other fluorescence channels to the possibility of extending the multiplexing for detection of other resistance genes, i.e., van-A/B, ESBL, or other carbapenemases type OXA, not the OXA-48 group.

Furthermore, the extraction-free protocol speeds up and facilitates the procedure. It can be easily applied to the routine as it requires minimal hands-on-time and can be further simplified with ready-to-use tubes, converting it into a good alternative for screening, in 24/7 clinical laboratories, in cases of sepsis and/or in patients with risk factors for being colonised by carbapenemase-producing bacteria.

Further multiplexing to detect ESBL, van A/B and other resistance genes within the same PCR tube would optimise the molecular screening of multi-resistant bacteria in our area. However, limitations of this assay for the expected application could be attributed to the possible deleterious effect on the efficiency of carbapenemase detection due to competitive factors when additional targets are investigated.

## 4. Material and Methods

### 4.1. Bacterial Strains and Samples

Strains of *bla*_OXA-181_-positive *Klebsiella pneumoniae*, *bla*_KPC-3_-positive *K. pneumoniae*, *bla*_VIM-1_-positive *Citrobacter freundii* complex, *bla*_NDM-5_-positive *K. pneumoniae*, and *bla*_IMP-23_-positive *Pseudomonas aeruginosa*, obtained from different clinical samples, were used for the design and optimisation of the real-time PCR assay. A clinical strain of *bla*_OXA-23_ *Acinetobacter baumannii* and a reference strain of *Escherichia coli* (ATCC^®^ 29212) were also included as negative controls during the optimisation procedure.

For specificity assays, 10 yeast and bacterial ATCC reference strains were used: *Candida tropicalis* (ATCC^®^ 760), *Issatchenkia orientalis* (ATCC^®^ 6258), *Streptococcus pneumoniae* (ATCC^®^ 49619), *Candida parapsilosis* (ATCC^®^ 22019), *P. aeruginosa* (ATCC^®^ 27853), *K. quasineumoniae* (ATCC^®^ 700603), *E. coli* (ATCC^®^ 29922), *E. coli* (ATCC^®^ 35218), *Enterococcus faecalis* (ATCC^®^ 29212) and *Staphylococcus aureus* (ATCC^®^ 29213).

For the analytical validation of the assay, 206 clinical isolates and 108 retrospective samples (91 rectal and 17 pharyngeal swabs) were used, in which the presence of the PCR-targeted carbapenemases had been previously confirmed by phenotypic methods in 189 and 36 cases, respectively (Table 4). Samples and strains were kept at −80 °C until use.

### 4.2. Diagnostic Methods

Rectal and pharyngeal swabs were routinely processed for surveillance of the presence of bacteria expressing KPC, OXA-48, IMP, VIM and NDM carbapenemases by culture in chromogenic CHROMID CPSO^®^ (bioMérieux, Marcy-L’Etoile, Francia) and/or selective media CHROMID ESBL^®^ (bioMérieux) [41].

Phenotypic-based methods to detect the carbapenemase enzymes included double disc synergy test and rapid, colourimetric, lateral-flow immunochromatographic assays: β CARBA test (Bio-Rad, Marnes-la-Coquette, France), NG Test^®^/CARBA-5 (NG Biotech, Guipry, Francia) and OXA-48 K-SeT (Coris Bioconcept, Gembloux, Belgium).

Identification of the carbapenemase-harbouring bacteria was carried out by MALDI-TOF mass spectrometry (Bruker Daltonics GmbH, Bremen, Germany). Vitek-2 system (bioMérieux) or Microscan (Beckman Coulter, Brea, CA, USA) and antibiotic gradient strips (bioMérieux or Liofilchem) were used for susceptibility testing in isolates with values above European Committee on Antimicrobial Susceptibility Testing (EUCAST) breakpoints for carbapenemase-producing bacteria screening.

The carbapenemase-producing type was confirmed by the Andalusian Laboratory of Molecular Typification of the Spanish Program for the Prevention and Control of Healthcare-related Infections and Appropriate Utilization of Antibiotics (acronym in Spanish, PIRASOA) by massive sequencing (Illumina Inc., San Diego, CA, USA), using CLC Genomics Workbench v10 (Qiagen, Shenzhen, China), ResFinder (Lyngby, Denmark) [https://cge.cbs.dtu.dk/services/ResFinder (accession date: 8 December 2024)], and CARD databases (Hamilton, ON, Canada) [(https://card.mcmaster.ca/ (accession date: 8 December 2024)]. Most bacterial strains in the present study were adequately characterised by different methods, determining whether they were nosocomial transmitted or transmission clusters, as previously described [41].

### 4.3. Design of the Real-Time PCR

#### 4.3.1. Primers and Probes

**Figure 2:**
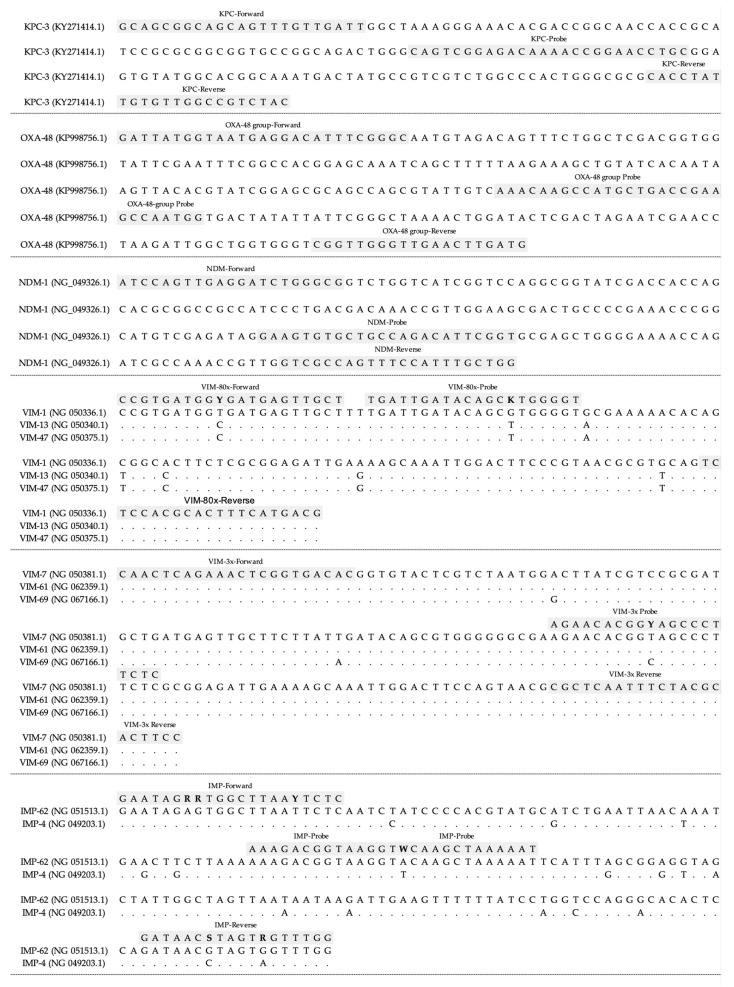
Nucleotide sequence of the fragment of each carbapenemase gene and the primers and probes used on these regions for the real-time PCR assay.

The published sequences of *bla*_KPC_, *bla*_IMP_, *bla*_VIM_, *bla*_NDM_ and *bla*_OXA-48-group_ carbapenemases available at GenBank [42] were obtained. MEGA 4 software [43] was used to align each carbapenemase gene with all the oligonucleotides selected for the real-time PCR, to check for sequence specificity (Figure 2).

Oligonucleotides used in the real-time PCR design process were obtained and/or checked for physicochemical properties and sequence specificity with Primer BLAST [44], PrimerQuest™ program (IDT, Coralville, IA, USA) and Primer-3 plus software [45]. Primers and Taqman^®^ probes for the amplification of internal control (human RNase P) were included in each PCR tube. The types and sequences of the different oligonucleotides used are displayed in Table 5.

#### 4.3.2. DNA Extraction

For the DNA extraction of bacterial isolates, suspensions at turbidity corresponding to a 0.5 McFarland scale were prepared. DNA was extracted from 200 µL of the suspension, spiked with 20 µL of human rhabdomyosarcoma cells (internal control) by heating in a thermal block at 98 °C for 10 min, and centrifuged at 14,000× *g* for 2 min. The supernatant was used as the DNA template.

For the DNA extraction from rectal samples, swabs were resuspended in Amies liquid medium tubes, vortexed and the suspensions were subjected to two DNA extraction processes:

- An automated extraction protocol in an OT-2 pipetting robot (Opentrons) using the Mag-Bind^®^ Viral DNA/RNA Xpress Kit (Omega Bio-Tek, Norcross, GA, USA) according to the manufacturer’s instructions.
- A DNA extraction-free protocol on rectal swabs, in which no sample processing was carried out apart from the vortexing step, prior to PCR.

Real-time PCR was carried out with 5 µL of samples (using the DNA extraction-free protocol) and/or purified DNA (from samples or bacterial isolates) in a total reaction volume of 20 µL on a CFX C1000 thermal cycler (Bio-Rad Laboratories, SA, Madrid, Spain).

#### 4.3.3. Real-Time PCR Conditions

To apply the molecular assay for surveillance purposes, the best PCR conditions were validated for a single-tube strategy to read VIM, IMP and/or NDM amplification on 6-FAM fluorescence channel and KPC and/or OXA-48 on HEX channel (Table 5).

Fluorescence was measured during the extension/annealing step of each cycle. Amplification of a fragment of human RNase P was used as internal control to confirm negative results when the cycle threshold (Ct) value was below 35.

Different master mixes were tested. The selection of the optimal one was completed according to three main parameters: Ct value, relative fluorescence units (RFU) and the amplification curve.

#### 4.3.4. Calculation of the Limit of Detection, Intra-Assay and Inter-Assay Coefficients of Variability

The LoD of the PCR for each carbapenemase target was calculated with six replicates of ten-fold serial dilutions of a 0.5 McFarland suspension of each carbapenemase-containing bacterial isolate. DNA extraction of each dilution was performed as described above. Undiluted bacterial suspensions and six replicates of the dilutions down to 10^−8^ were tested with the real-time PCR. A 10 µL aliquot of each bacterial suspension and dilution was subcultured in blood agar plates to carry out the real CFU count and correlate it with the real-time PCR positivity.

The LoD was defined as the lowest dilution, expressed in CFU/reaction that gave a positive result in the six replicates.

Intra-assay and inter-assay coefficients of variability were calculated with five replicates of each bacterial dilution within the same PCR run and with aliquots of the same replicates tested in different PCR runs, respectively. The average, standard deviation (SD) and coefficient of variation (CV) of the Ct were calculated.

A linear regression analysis was carried out and the correlation coefficient (R2) was calculated.

**Figure 3:**
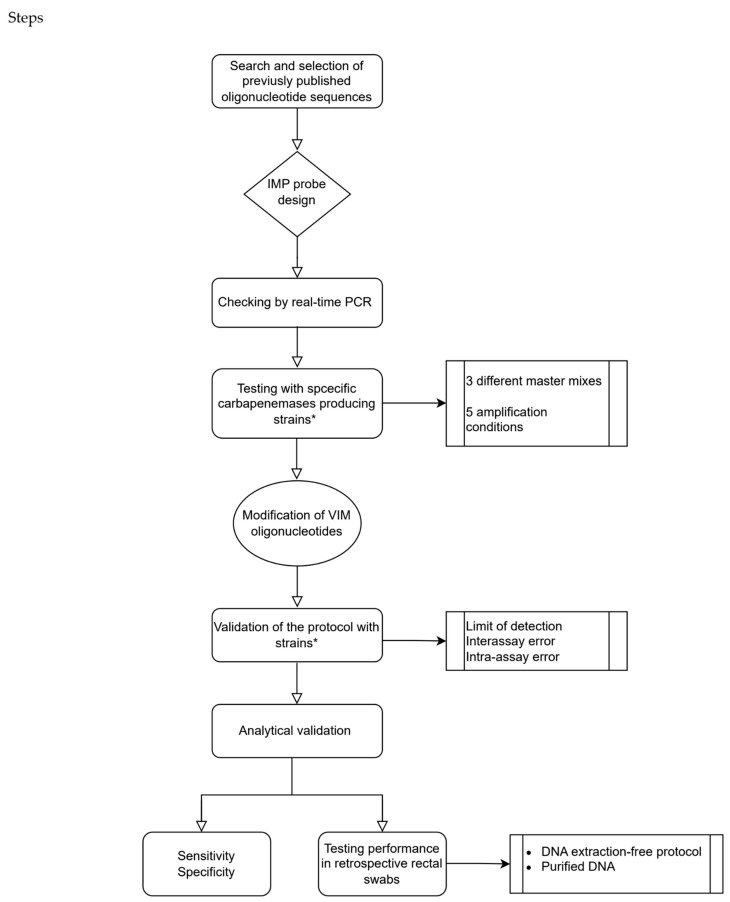
Schematic workflow of the procedures carried out for the design, optimisation and validation of the real-time PCR. * See Section 4.1 (bacterial strains and samples).

Figure 3 displays a scheme of the steps followed for the design, optimisation and validation of the real-time PCR.

### 4.4. Statistical Analysis

Qualitative and quantitative results were recorded in an MS Excel database, and descriptive analysis was carried out.

### 4.5. Ethics Approval and Consent to Participate

The study protocol was conducted in accordance with the Declaration of Helsinki and the ethical considerations of epidemiological research. This was a non-interventional study, with no further investigation into routine procedures. The biological material was only used for the standard diagnosis as ordered by attending physicians. No additional sampling or modification of the routine diagnostic protocol was used. Data analyses were performed using a completely anonymous database, where subjects were replaced by different infectious episodes, occurring at least 6 weeks apart from the previous one, if any. Permission to access and use the data was granted by the Clinical Microbiology Management Unit of Virgen de las Nieves University Hospital (Granada, Spain) and Clinical Microbiology Management Unit of Málaga Regional University Hospital. Ethics committee approval was considered unnecessary according to national guidelines [Law on Data Protection-Organic Law 15/1999 of December 13 on the protection of data of a personal nature, https://www.boe.es/eli/es/lo/1999/12/13/15, accession date: 15 January 2020).

## 5. Conclusions

The real-time PCR assay offers a rapid, sensitive, and specific method for detecting major carbapenemase genes. It provides an effective tool for surveillance and infection control in clinical settings allowing for targeted interventions and limiting the spread of multidrug-resistant organisms. The extraction-free protocol converts this method into a good alternative for screening in 24/7 clinical laboratories. Furthermore, the design of the multiplex PCR allows for potential expansion to include additional carbapenemase or other resistance genes in the same tube, on demand, depending on the clinical setting.

## Figures and Tables

**Table 1:** Primers/probe sets combinations, amplification conditions and master mixes tested for the design of the real-time PCR.

Concentration of Primers and Probes (µM)			Amplification Protocols	
Primers/Probe Set	Primers	Probe	Name	PCR Conditions
VIM	0.5	0.2	P1	60 °C/1 min
	0.75	0.3		95 °C/10 min
	1	0.4		45 cycles: 95 °C/15 s
	1.5	0.6		58 °C/60 s
IMP	0.5	0.2		
	1	0.4		
NDM	0.4	0.15	P2	50 °C/10 min
	0.5	0.2		95 °C/1–3 min
	0.75	0.3		45 cycles: 95 °C/10 s
	1	0.4		58–60 °C/40 s
KPC	0.5	0.2		
	1	0.4		
OXA-48	0.5	0.2	P3	95 °C/1–3 min
	1	0.4		45 cycles: 95 °C/15 s
RNase P	0.25	0.1		50 °C/30–40 s
	0.6	0.3		72 °C/20–60 s
**Master mixes**				
			P4	95 °C/1–3 min
				45 cycles: 95 °C/10 s
Quantabio qScriptXLT 1-Step RT-qPCR ToughMix (Quantabio)				60 °C/40–60 s
	
LightCycler FastStart DNA Master ^PLUS^ (Roche Diagnostics)			P5	95 °C/3 min
TaqPath™ 1-Step Multiplex Master Mix (No ROX) (Thermofisher, Waltham, MA, USA)				45 cycles: 95 °C/10 s
				50 °C/30 s
				60 °C/30 s

**Table 2:** Intra-assay and inter-assay variability of the real-time PCR assay.

Carbapenemase	Intra-Assay Variability				Inter-Assay Variability			
	Dilution ^1^	Mean Ct	SD	CV	Dilution ^1^	Mean Ct	SD	CV
VIM	10^−2^	29.47	0.81	2.74	10^−1^	26.82	1.02	3.79
					10^−2^	29.37	0.96	3.26
					10^−3^	32.26	1.25	3.87
IMP	10^−2^	25.19	0.64	2.55	10^−1^	22.57	0.81	3.61
					10^−2^	25.87	0.81	3.14
					10^−3^	30.56	0.80	2.61
NDM	10^−2^	31.13	0.61	1.97	10^−1^	26.62	1.13	4.25
					10^−2^	31.20	0.87	2.78
					10^−3^	34.05	0.63	1.85
KPC	10^−2^	26.61	0.89	3.34	10^−1^	23.61	0.99	4.19
					10^−2^	28.09	1.73	6.17
					10^−3^	30.67	1.01	3.31
OXA-48	10^−2^	26.13	0.26	0.99	10^−1^	24.14	0.82	3.41
					10^−2^	25.62	0.76	2.96
					10^−3^	31.15	1.68	5.40

^1^ Dilutions starting from 0.5 McFarland suspensions.

**Table 3:** Results of real-time PCR and phenotypic assays in the 108 retrospective samples.

DNA Template	Phenotypic Results (*n*)							
	OXA-48 (8)	IMP (3)	VIM (10)	NDM (11)	KPC + VIM (1)	OXA-48 + NDM (2)	NDM + KPC (1)	Other Resistance Genes (72)
Purified DNA								
Negative	-	-	-	-	-	-	-	71
Positive KPC/OXA-48 group	8/8	-	-	-	-	-	-	-
Positive MBL	-	3/3	10	9	-	-	-	-
Positive MBL and KPC/OXA-48	-	-	-	2 *	1	2	1	1
Extraction-free DNA								
Invalid	-	-	-	1	-	-	-	-
Negative	-	-	-	-	-	-	-	71
Positive KPC/OXA-48 group	8	-	-	-	-	-	-	-
Positive MBL	-	3	10	8	-	-	-	-
Positive MBL and KPC/OXA-48	-	-	-	2 *	1	2	1	1

* Additional KPC/OXA-48 genes were detected by molecular assay with both DNA templates in two samples.

**Table 4:** Bacterial isolates and retrospective samples used for the analytical validation of the real-time PCR assay.

	KPC	OXA-48	IMP	VIM	NDM	KPC + VIM	OXA-48 + NDM	OXA-48 + VIM	NDM + KPC	Other Resistance Genes
Bacterial isolates (206)										
*Klebsiella pneumoniae* (93)	11	60	1	2	16	1	2	-	-	-
*Klebsiella oxytoca* (7)	1	-	-	5	-	-	1	-	-	-
*Citrobacter freundii* (7)	-	1	1	2	1	-	-	1	-	1
*Citrobacter amalonaticus* (1)	-	-	-	1	-	-	-	-	-	-
*Escherichia coli* (14)	-	7	-	3	3	-	-	-	1	-
*Pseudomonas aeruginosa* (52)	-	-	33	9	-	-	-	-	-	9
*Pseudomonas putida* (2)	-	-	-	2	-	-	-	-	-	-
*Enterobacter cloacae* complex (25)	-	4	-	20	-	-	-	-	-	1
*A. baumannii* complex (6)	-	-	-	-	-	-	-	-	-	6
Swab samples (108)	-	8	3	10	11	1	2	0	1	72

**Table 5:** Primers and probes used in the study for the design and validation of an in-house real-time PCR assay for the detection of *bla*_KPC_, *bla*_IMP_, *bla*_VIM_, *bla*_NDM_ and *bla*_OXA-48-group_ carbapenemases.

Target	Primer/Probe Name	Sequence (5′-3′)	Source
IMP	IMP-Forward	GAA TAG RRT GGC TTA AYT CTC	[46]
	IMP-Reverse	CCA AAC YAC TAS GTT ATC	[46]
VIM	VIM-80X-Forward	CCG TGA TGG YGA TGA GTT GCT	This study
	VIM-3X-Forward	CAA CTC AGA AAC TCG GTG ACA C	This study
	VIM-80X-Reverse	CGT CAT GAA AGT GCG TGG AGA	This study
	VIM-3X-Reverse	GGA AGT GCG TAG AAA TTG AGC G	This study
NDM	NDM-Forward	ATC CAG TTG AGG ATC TGG GCG	[47]
	NDM-Reverse	CCA GCA AAT GGA AAC TGG CGA C	[47]
KPC	KPC-Forward	GCA GCG GCA GCA GTT TGT TGA TT	[47]
	KPC-Reverse	GTA GAC GGC CAA CAC AAT AGG TGC	[47]
OXA-48	OXA-48-Forward	GAT TAT GGT AAT GAG GAC ATT TCG GGC	[47]
	OXA-48-Reverse	CAT ATC CAT ATT CAT CGC AAA AAA CCA CAC	[47]
RNase P	RNase P-Forward	AGA TTT GGA CCT GCG AGC G	CDC *
	RNase P-Reverse	GAG CGG CTG TCT CCA CAA GT	CDC *
*Taqman^®^ probes*			
IMP	IMP-HEX	HEX-AAG ACG GTA AGG TWC AAG CTA AAA AT-BHQ1	This study
VIM	VIM-80X-HEX	HEX-TGA TTG ATA CAG CKT GGG GT-BHQ1	This study
	VIM-3X-HEX	HEX-AGA ACA CGG YAG CCC TTC TC-BHQ1	This study
NDM	NDM-HEX	Texas Red-ACC GAA TGT CTG GCA GCA CAC TTC-BHQ2	[47]
KPC	KPC-FAM	FAM-CAG TCG GAG ACA AAA CCG GAA CCT GC-BHQ1	[47]
OXA-48	OXA-48-FAM	HEX-CCA TTG GCT TCG GTC AGC ATG GCT TGT TT-BHQ1	[47]
RNase P	RNase P-Cy5	Cy5-TTC TGA CCT GAA GGC TCT GCG CG-BHQ3	CDC *

* https://www.cdc.gov/coronavirus/2019-ncov/lab/multiplex.html (accession date: 15 January 2020).

## Data Availability

The original contributions presented in this study are included in the article. Further inquiries can be directed to the corresponding author.
